# Synergistic Clinico-Radiological Assessment of Term Newborns Afflicted by Birth Asphyxia in a Tertiary Medical Center: A Prospective Observational Study

**DOI:** 10.7759/cureus.73321

**Published:** 2024-11-09

**Authors:** Virinchi Jampala, Vasudev Kompally, Subhan B Bukkapatnam, Pratap Gudi

**Affiliations:** 1 Department of Pediatrics, Government Medical College, Jayashankar Bhupalpally, Bhupalpally, IND; 2 Department of Pediatrics, Gandhi Medical College, Secunderabad, IND; 3 Department of Pediatrics, Government Medical College, Narsampet, Narsampet, IND

**Keywords:** electroencephalography, hypoxic-ischemic encephalopathy, neonatal outcomes, neuroimaging, therapeutic hypothermia

## Abstract

Background and objectives

Hypoxic-ischemic encephalopathy (HIE) remains a critical concern in newborns, with therapeutic hypothermia (TH) serving as a key treatment strategy. However, assessing long-term outcomes requires a comprehensive approach, as children may develop neuropsychological and learning challenges even in the absence of noticeable motor impairments. This study aims to explore the correlation between electroencephalography (EEG) and neuroimaging findings with the clinical severity of HIE.

Materials and methods

This prospective observational study included a sample of 110 term neonates with birth asphyxia admitted to a medical college-affiliated hospital in Telangana, India. Data were collected on a predesigned Case Record Form by a single investigator through direct interviews with parents, caregivers, and other relevant individuals.

Results

Among the 110 neonates analyzed, most fell into HIE Grade II (49.1%), delivered predominantly via normal vaginal delivery (61.8%). Common risk factors included prolonged labor (57.3%) and meconium-stained amniotic fluid (42.7%). Serum parameters revealed variations, with significant associations between sodium, potassium, blood urea, blood culture, EEG, and magnetic resonance imaging (MRI) findings and HIE staging. Abnormal EEG (36.4%) and MRI (63.6%) findings correlated significantly with HIE severity.

Conclusions

The study provides valuable insights into clinico-radiological correlations in term newborns with birth asphyxia, highlighting the diagnostic value of EEG and neuroimaging modalities. Serum parameters and imaging findings showed significant associations with HIE severity, emphasizing the importance of comprehensive assessment in predicting neonatal outcomes. EEG and MRI demonstrated high sensitivity in predicting abnormal neurological conditions at discharge, suggesting their utility in prognostication.

## Introduction

Neonatal and perinatal mortality rates are pivotal indicators of a nation's health status, reflecting the effectiveness of healthcare systems in safeguarding the most vulnerable members of society. While developed countries typically boast lower mortality rates, India faces a significant challenge, with its neonatal mortality rate (NMR) standing at 22 per 1,000 live births as of 2019. This rate considerably surpasses the global average, highlighting pronounced disparities between urban and rural regions. In Telangana specifically, the NMR stands at 20 per 1,000 live births, reflecting significant public health concerns in the region. Notably, prematurity accounts for a substantial portion, contributing to 35% of neonatal deaths, according to the Health and Family Welfare Statistics in India 2019-2020 [1].

Hypoxic-ischemic encephalopathy (HIE) presents a formidable threat to neonatal health, affecting approximately 1-6 neonates per 1,000 births in developed nations. If left untreated, HIE can result in death in 15-20% of cases, with an additional 25% facing significant disability [2]. However, the introduction of therapeutic hypothermia (TH) treatment has heralded a promising shift, leading to a reduction in mortality and morbidity rates associated with HIE. Given that HIE symptoms typically emerge within the first days of life, timely and accurate assessment becomes paramount for initiating appropriate treatment and predicting long-term outcomes [3]. To this end, a range of diagnostic tools - including neurological assessments, neuroimaging, and continuous electroencephalography (EEG) - are employed to monitor symptom progression, with the severity of encephalopathy often assessed using established classification systems such as the Sarnat and Sarnat classification [4].

The understanding of childhood outcomes following neonatal HIE necessitates comprehensive assessment protocols that consider the timing and methodology of neurodevelopmental evaluations. Despite efforts to categorize outcomes by HIE grade, the prognosis for moderate HIE remains variable due to its heterogeneous nature. While mild HIE outcomes were traditionally viewed as benign, recent consensus underscores the need for further investigation into their long-term implications. Consequently, infants with moderate to severe neonatal HIE were predominantly included in TH randomized controlled trials during the early 2000s [5].

To advance our understanding of neonatal and long-term trajectories, ongoing monitoring of all HIE grades into school age is imperative to capture the nuanced patterns of development. Given that TH treatment decisions are often made within six hours of birth, early assessment tools, such as EEG, continue to play a crucial role in prognostication, highlighting the importance of timely intervention in improving neonatal outcomes.

The aim of this study is to correlate EEG and neuroimaging, such as cranial ultrasonography (CUS) and magnetic resonance imaging (MRI) changes, with the clinical severity of HIE, with the objective of evaluating the immediate outcomes.

## Materials and methods

This study is a prospective observational study conducted in the Department of Pediatrics, Kakatiya Medical College, and Mahatma Gandhi Memorial (MGM) Hospital, Warangal, India. The research was carried out between January 2021 and November 2022, following ethical clearance (KMC/IEC/Paed/2020-008). Informed consent was obtained from the parents of all neonates enrolled in the study. The study aimed to evaluate clinicoradiological correlations in term neonates with birth asphyxia.

The inclusion criteria for the study comprised term neonates with a history of delayed cry at birth, who were revived following Neonatal Resuscitation Program (NRP) guidelines, and were admitted to the Neonatal Intensive Care Unit (NICU) of MGM Hospital. Only hemodynamically stable neonates diagnosed with HIE in stages 1, 2, or 3 were included.

The exclusion criteria involved hemodynamically unstable neonates, preterm babies, those with congenital anomalies or metabolic disorders, and neonates with hyperbilirubinemia leading to kernicterus.

A total of 110 term neonates meeting the eligibility criteria were recruited during the study period. Data collection involved assessing gestational age (GA) using the Ballard scoring system, which evaluates physical and neurological maturity, along with confirmation based on the mother’s last menstrual period (LMP). The severity of HIE was evaluated using the Sarnat and Sarnat grading system, which classifies neonates into three stages based on clinical and neurological signs.

Neuroimaging techniques, including CUS and MRI, were employed to evaluate the extent of brain injury. MRI was conducted within four to seven days of birth to detect abnormalities, while CUS was used as a preliminary screening tool to guide further imaging. Additionally, EEG was performed to monitor seizures and assess the electrical activity of the brain.

Several blood investigations were carried out, including blood cultures to detect infections, and tests for sodium, potassium, random blood sugar (RBS), urea, and serum creatinine levels to evaluate the metabolic status of the neonates. The findings from MRI, EEG, and clinical evaluations were correlated with the HIE stages to determine the severity and potential outcomes.

Statistical analysis

Data were recorded in a pre-designed Case Record Form and entered into Microsoft Excel (Microsoft® Corp., Redmond, WA, USA). Statistical analyses were performed using IBM SPSS Statistics for Windows, Version 26 (Released 2019; IBM Corp., Armonk, NY, USA). Descriptive statistics, including means, standard deviations, frequencies, and percentages, were calculated for continuous and categorical variables. The Chi-square test was used to evaluate associations between categorical variables, with significance determined at p < 0.05. Results were visually represented through bar charts and pie charts to enhance clarity.

The study also evaluated the neurological condition of neonates at the time of discharge and analyzed the predictive value of MRI and EEG in assessing neurological outcomes. Sensitivity, specificity, positive predictive value (PPV), and negative predictive value (NPV) were calculated to determine the effectiveness of these diagnostic tools.

## Results

During the two-year study period, a total of 110 term neonates with birth asphyxia were analyzed. For categorical variables, such as delivery type and risk factors, we used the Chi-square test, which showed a significant association between delivery type and HIE grades (χ²(4, N = 110) = 12.8, p = 0.02). Additionally, logistic regression analysis was performed to predict the likelihood of abnormal neurological outcomes at discharge based on MRI and EEG findings. The results demonstrated that abnormal EEG findings significantly increased the odds of poor outcomes (OR = 2.5, 95% CI: 1.4-4.2, p < 0.001), while MRI abnormalities further elevated this risk (OR = 3.2, 95% CI: 1.8-5.6, p < 0.001).

Table 1 presents the distribution of subjects according to HIE grading, with the majority categorized as Grade 2. Table 1 also outlines the prevalence of risk factors, with prolonged labor being the most common. We used Spearman's rank correlation coefficient to look for links between continuous variables. It showed a moderately positive link (ρ = 0.42, p < 0.001) between potassium levels and neurological problems. These additional statistical analyses provide a more comprehensive understanding of the relationships between clinical parameters and outcomes, underscoring the importance of EEG, MRI, and biochemical markers in predicting neonatal prognosis.

**Table 1:** Type of delivery and risk factors Chi-square test, which showed a significant association between delivery type and HIE grades (χ²(4, N = 110) = 12.8, p = 0.02). LSCS: Lower segment cesarean section; MSAF: Meconium-stained amniotic fluid; APH: Antepartum hemorrhage; GDM: Gestational diabetes mellitus; HIE: Hypoxic-ischemic encephalopathy

Type of delivery	No.	Percent (%)
Normal vaginal	68	61.8
Assisted vaginal	4	3.6
LSCS	38	34.5
Risk factors		
Prolonged labor	63	57.3
MSAF	47	42.7
Cord around neck	14	12.7
Oligohydramnios	12	10.9
APH	9	8.2
GDM	4	3.6
Preeclampsia	4	3.6
None	14	12.7

Table 2 depicts the distribution of subjects based on various clinical parameters, including sodium, potassium, RBS, blood urea, serum creatinine, and blood culture, revealing significant correlations, particularly with sodium, potassium, blood urea, and blood culture.

**Table 2:** HIE staging and lab characteristics A one-way ANOVA was applied to compare continuous variables, such as sodium, potassium, and blood urea levels, across the three HIE stages (I, II, and III), which revealed significant differences among the groups (e.g., sodium: F(2,107) = 5.89, p = 0.004). Post-hoc Tukey tests indicated a significant difference in sodium levels between HIE Grades II and III (p = 0.01). Note: This study population has not shown hypokalemia. DOL: Day of life; GRBS: Glucose random blood sugar; CoNS: Coagulase-negative staphylococcus; ANOVA: Analysis of variance; HIE: Hypoxic-ischemic encephalopathy

	Characteristic	HIE staging			p-value
		I (n = 10)	II (n = 54)	III (n = 46)	
DOL	Day 1	38 (34.5)	65 (59.1)	7 (6.4)	-
	Day 2	15 (13.6)	52 (47.3)	43 (39.1)	
	Day 3	10 (9.1)	54 (49.1)	46 (41.8)	
GRBS	≤140	7 (70.0)	43 (79.6)	36 (78.3)	0.172
	140-200	2 (20.0)	3 (5.6)	8 (17.4)	
	>200	1 (10.0)	8 (14.8)	2 (4.3)	
Sodium	Hyponatremia	-	5 (9.3)	15 (32.6)	0.003
	Normal	10 (100.0)	44 (81.5)	31 (67.4)	
	Hypernatremia	-	5 (9.3)	-	
Potassium	Normal	10 (100.0)	48 (88.9)	34 (73.9)	0.044
	Hyperkalemia	-	6 (11.1)	12 (26.1)	
Blood urea	Normal	10 (100.0)	42 (77.8)	28 (60.9)	0.021
	Abnormal	-	12 (22.2)	18 (39.1)	
Blood culture	Acinetobacter	-	14 (25.9)	8 (17.4)	0.008
	Klebsiella	-	9 (16.7)	6 (13.0)	
	Pseudomonas	-	3 (5.6)	-	
	CoNS	-	1 (1.9)	1 (2.2)	
	Citrobacter	-	-	1 (2.2)	
	Sterile	10 (100.0)	27 (50.0)	30 (65.2)	

Additionally, we performed logistic regression analysis to predict the likelihood of abnormal neurological outcomes at discharge based on MRI and EEG findings. The results demonstrated that abnormal EEG findings significantly increased the odds of poor outcomes (OR = 2.5, 95% CI: 1.4-4.2, p < 0.001), and MRI abnormalities further increased the risk (OR = 3.2, 95% CI: 1.8-5.6, p < 0.001). Table 3 shows associations between EEG, neurosonography (NSG), and MRI findings and HIE staging, revealing significant correlations between EEG and MRI.

**Table 3:** Radiological features association with HIE staging Abnormal EEG findings significantly increased the odds of poor outcomes (OR = 2.5, 95% CI: 1.4-4.2, p < 0.001), and MRI abnormalities further increased the risk (OR = 3.2, 95% CI: 1.8-5.6, p < 0.001). EEG: Electroencephalography; MRI: Magnetic resonance imaging; NSG: Neurosonography; HIE: Hypoxic-ischemic encephalopathy

	Characteristic	HIE staging			p-value
		I (n = 10)	II (n = 54)	III (n = 46)	
NSG	Normal	10 (100.0)	51 (94.4)	37 (80.4)	0.041
	Abnormal	-	3 (5.6)	9 (19.6)	
EEG	Normal	10 (100.0)	41 (75.9)	19 (41.3)	<0.001
	Abnormal	-	13 (24.1)	27 (58.7)	
MRI	Normal	9 (90.0)	26 (48.1)	5 (10.9)	<0.001
	Abnormal	1 (10.0)	28 (51.9)	41 (89.1)	

The Chi-square test conducted on the data for neurological condition at discharge resulted in a p-value of 1.0. This indicates that the observed distribution between normal and abnormal neurological conditions at discharge does not differ significantly from what would be expected by chance. With the p-value exceeding the conventional threshold of 0.05, it suggests that the difference in clinical outcomes (normal vs. abnormal) is not statistically significant within the analyzed sample. Additionally, Table 4 highlights the neurological condition at discharge, with the majority having a normal neurological condition.

**Table 4:** Neurological condition at discharge The Chi-square test conducted on the data for the neurological condition at discharge resulted in a p-value of 1.0.

Clinical condition	Number	Percent (%)
Normal	62	56.4
Abnormal	48	43.6

To assess correlations between continuous variables, we applied Spearman’s rank correlation coefficient, which indicated a moderate positive correlation between potassium levels and abnormal neurological outcomes (ρ = 0.42, p < 0.001). The correlation between neurological outcomes at discharge (normal vs. abnormal) and findings from EEG, MRI, and a combined EEG + MRI approach was also examined. EEG shows a sensitivity of 62.5% and specificity of 83.7%, with a PPV of 75.0% and an NPV of 74.3%. MRI findings exhibit higher sensitivity (83.3%) but lower specificity (51.6%), with a PPV of 57.1% and NPV of 80.0%. The combined EEG + MRI approach provides the highest sensitivity (93.8%) but with reduced specificity (48.4%), yielding a PPV of 58.4% and an NPV of 90.9%. All associations are statistically significant, with p-values <0.001. These findings underscore the importance of comprehensive clinical assessment and diagnostic imaging in predicting neonatal outcomes (Table 5).

**Table 5:** Radiological correlation with neurological outcome EEG shows a sensitivity of 62.5% and specificity of 83.7%, with a positive predictive value (PPV) of 75.0% and a negative predictive value (NPV) of 74.3%. MRI findings exhibit higher sensitivity (83.3%) but lower specificity (51.6%), with a PPV of 57.1% and NPV of 80.0%. The combined EEG + MRI approach provides the highest sensitivity (93.8%) but with reduced specificity (48.4%), yielding a PPV of 58.4% and an NPV of 90.9%. All associations are statistically significant, with p-values <0.001. #PPV: Positive predictive value; *NPV: Negative predictive value EEG: Electroencephalography; MRI: Magnetic resonance imaging

		Neurological condition at discharge		p-value	Sensitivity	Specificity	PPV^#^	NPV^*^
		Abnormal	Normal					
EEG	Abnormal	30	10	<0.001	62.50%	83.70%	75.00%	74.30%
	Normal	18	52					
MRI	Abnormal	40	30	<0.001	83.30%	51.60%	57.10%	80.00%
	Normal	8	32					
EEG + MRI	Abnormal	45	32	<0.001	93.80%	48.40%	58.40%	90.90%
	Normal	3	30					

## Discussion

The prospective study conducted at MGM Hospital from January 2021 to November 2022 focused on the clinico-radiological correlation in term newborns with birth asphyxia. A sample of 110 term babies with HIE was analyzed, examining parameters such as the type of delivery, risk factors, and various serum and imaging markers.

In terms of delivery, our study found that 61.8% were delivered by normal vaginal delivery (NVD) and 34.5% by lower segment cesarean section (LSCS). HIE grades were distributed as follows: 9.1% in Grade I, 49.1% in Grade II, and 41.8% in Grade III. Risk factors such as prolonged labor, meconium-stained amniotic fluid (MSAF), and cord around the neck were observed, with 12.7% showing no complications.

Serum parameters revealed variations compared to other studies [6,7]. For instance, hypernatremia and hyperkalemia were observed less frequently, at 18.2% and 15.5%, respectively. Blood urea and serum creatinine levels were also lower in our study compared to others [8]. Similarly, the prevalence of hypoglycemia was lower, at 21.8%.

Imaging studies, including EEG, NSG, and MRI, highlighted abnormalities consistent with HIE. EEG abnormalities were observed in 36.4% of subjects, while NSG and MRI abnormalities were seen in 10.9% and 36.4%, respectively. MRI showed a higher prevalence of white matter injury (28.2%) compared to grey matter injury (25.4%) [9,10].

Correlations between EEG, NSG, and MRI findings and HIE severity were significant, indicating the diagnostic value of these modalities. Notably, abnormal MRI findings correlated with severe HIE grades, with 48.23% of Grade 3 cases showing abnormalities [10,11].

At discharge, 56.4% of subjects exhibited normal neurological conditions, suggesting favorable outcomes compared to other studies. The results of our study regarding the diagnostic and prognostic significance of imaging techniques in HIE in neonates are consistent with findings from several previous studies, which also emphasize the critical role of neuroimaging in managing HIE. Giri et al. (2020) conducted a comparative study on transcranial ultrasound and MRI to assess the imaging characteristics of HIE in clinically diagnosed cases, providing insights into the diagnostic efficacy of each modality [12]. Priyanka et al. (2017) expanded on this work by investigating the association between MRI patterns of HIE in term infants and corresponding neurosonographic features. Their findings emphasized that MRI abnormalities, particularly signal irregularities in critical areas such as the basal ganglia and the posterior limb of the internal capsule, exhibit a strong correlation with clinical outcomes, underlining MRI’s prognostic value in HIE [13]. Pierrat et al. (2005) contributed an epidemiological perspective, examining the prevalence, causative factors, and outcomes of neonatal encephalopathy. Their study illustrated how the etiology of encephalopathy, including HIE, significantly influences clinical progression and prognosis, thus emphasizing the importance of understanding underlying causes for effective management [14]. Finally, Mcintyre et al. (2015) explored the impact of various etiologies of neonatal encephalopathy on treatment responses and outcomes, highlighting the differential effects of causative factors on therapeutic success [15]. These observations are consistent with our own study, where certain imaging patterns were indicative of poorer neurodevelopmental outcomes. Their work highlights the importance of MRI as a tool not only for diagnosing HIE but also for predicting neurological outcomes, reinforcing our approach of using advanced imaging modalities for more accurate prognostication. Associations between EEG, MRI, and neurological conditions at discharge were statistically significant, with combined EEG and MRI showing high sensitivity (93.8%) and moderate specificity (48.4%).

Overall, the study provides valuable insights into the clinico-radiological correlation in term newborns with birth asphyxia, highlighting the importance of early diagnosis and intervention to improve outcomes in these cases.

Limitations

This study has several limitations. The single-center design may limit the generalizability of our findings. Additionally, the relatively small sample size could impact the statistical power of some analyses. Further, multicenter studies with larger cohorts are needed to validate these findings and explore regional variations in HIE management and outcomes.

Future directions

Future research should focus on expanding these findings through larger, multicentric studies to validate the prognostic value of EEG and MRI in diverse populations. Additionally, exploring the impact of early therapeutic interventions based on diagnostic findings could provide further insights into optimizing treatment protocols for HIE. Continued investigation into the long-term neurodevelopmental outcomes of neonates with varying grades of HIE will be crucial for refining management strategies and improving overall neonatal care.

## Conclusions

The study provides valuable insights into the clinico-radiological correlations of term neonates with birth asphyxia, emphasizing the importance of comprehensive assessment using EEG and MRI. The significant associations between serum parameters, imaging findings, and HIE severity highlight the need for an integrated diagnostic approach to predict and manage neonatal outcomes effectively. The high sensitivity of combined EEG and MRI in predicting abnormal neurological conditions at discharge supports their use in early prognostication and intervention. Ongoing monitoring and tailored management strategies are essential for improving long-term outcomes for neonates affected by birth asphyxia.
